# Evaluation of autologous platelet rich plasma for cardiac surgery: outcome analysis of 2000 patients

**DOI:** 10.1186/s13019-016-0452-9

**Published:** 2016-04-12

**Authors:** Amit N. Patel, Craig H. Selzman, Ganesh S. Kumpati, Stephen H. McKellar, David A. Bull

**Affiliations:** Division of Cardiothoracic Surgery, University of Utah School of Medicine, Salt Lake City, UT 84132 USA

**Keywords:** Cardiac surgery, Wounds, Sternal, Infections, Platelets

## Abstract

**Background:**

Deep and superficial sternal wound infections (DSWI & SWI) following cardiac surgery increase morbidity, mortality and cost. Autologous platelet rich plasma (PRP) derived from the patient’s own blood has been used in other surgical settings to promote successful wound healing. The goal of this study was to analyze the addition of PRP using a rapid point of care bedside system to standard wound care in all patients undergoing sternotomy for cardiac surgical procedures.

**Methods:**

Over a 7 year period, 2000 patients undergoing open cardiac operations requiring sternotomy were enrolled. One thousand patients received standard of care sternal closure. The other 1000 patients received standard of care sternal closure plus PRP applied to the sternum at the time of closure. The outcomes related to wound healing, infection, readmissions, and costs were analyzed.

**Results:**

In the 2000 patients, there were more ventricular assist device implants/heart transplants and emergency operations in the PRP group; otherwise there were no significant differences. The use of PRP reduced the incidence of DSWI from 2.0 to 0.6 %, SWI from 8.0 to 2.0 %, and the readmission rate from 4.0 to 0.8 %. The use of PRP reduced the costs associated with the development of deep and superficial wound complications from $1,256,960 to $593,791.

**Conclusions:**

The use of PRP decreases the incidence and costs of sternal wound complications following cardiac surgery. The routine use of platelet rich plasma should be considered for all patients undergoing sternotomy for cardiac surgical procedures.

**Trial registration:**

Clinicaltrials.gov (NCT00130377) for the data registry.

## Background

Sternal wound complications such as dehiscence and infection following median sternotomy occurs in 0.2 to 8 % of patients undergoing cardiac surgery [1]. These post-surgical complications remain a significant source of mortality, and treatment entails extended hospitalization, long-term antibiotics, multiple operative procedures, and high cost. Several patient and surgical related risk factors for the development of sternal wound complications have been identified [2, 3]: obesity, bilateral harvesting of the internal mammary artery, diabetes, steroid treatment, advanced age, active smoking, osteoporosis, and chronic lung disease. Several therapeutic strategies have been used to minimize the incidence of deep sternal wound infections (DSWI) in high-risk patients [4]. However, once a DSWI occurs, debridement, continuous antibiotic irrigation, and vacuum-assisted closure therapy procedures have decreased the incidence of DSWI related mortality [5–7]. A preventative therapeutic strategy has yet to be clearly identified. The topical application of autologous platelet rich plasma (PRP) to promote earlier wound healing in a variety of settings has been described [8]. Several recent reports have demonstrated the benefits of using the topical application of PRP for improved postoperative outcomes following median sternotomy [9–11]. Most of these studies, however, have been under-powered and have no financial analysis of the potential cost savings associated with the use of PRP therapy. Currently, the Centers for Medicaid and Medicare Services do not reimburse for the costs incurred during the treatment of DSWI. Metrics such as quality now help determine reimbursement in the value based system of healthcare. Our goal in this outcomes study was to determine the incidence and costs of sternal wound complications in 1000 consecutive patients undergoing sternotomy for cardiac surgery procedures that received PRP and compare them to 1000 patients that did not receive PRP.

## Methods

### Patients

**Fig. 1:**
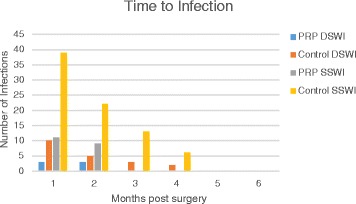
Time to infection post-surgery. The time to infection post-surgery was reported as number of patients with infections. PRP DSWI- platelet rich plasma deep sternal wound infection, Control DSWI – control with deep sternal wound infection, PRP SSWI- platelet rich plasma superficial sternal wound infection, Control SSWI – control with superficial sternal wound infection

Between January 2005 and January 2013, 2000 consecutive patients undergoing open cardiac surgery requiring a sternotomy at a single cardiac surgical center were consented and were included in our database (NCT00130377). University of Utah IRB approval #35242. The data was also prospectively collected as part of the Society of Thoracic Surgeons database at our institution. There were 1000 consecutive patients who received standard of care sternal closure plus PRP composed of autologous platelet rich plasma, calcium and thrombin applied to the sternum at the time of closure from December 2009 to January 2013 (PRP). These patients were compared to the previous one thousand consecutive patients who received standard of care sternal closure, including preoperative antibiotics and protocol driven glycemic management from January 2005 to November 2009 (Control). All data was prospectively collected and retrospectively analyzed. The outcomes related to deep and/or superficial sternal wound infections, readmissions, and actual costs were analyzed for 6 months post-surgery (Fig. 1). Sternal wound infections were characterized by the Centers for Disease Control and Prevention (CDC) definitions: superficial, deep, or organ/space surgical site infections [12]. All patients received standardized preoperative intravenous antibiotics within 60 min of the procedure. The preoperative antibiotics protocol and glycemic management protocol were standard and remained the same for all patients throughout the study. Standard surgical methods were used for the performance of all median sternotomies and sternal closures. All patients undergoing sternotomy were included in the study including emergencies, reoperations, ventricular assist device implantations, heart transplants, aortic dissections and standard operations (i.e. coronary artery bypass grafting and valve repairs or replacements). Matching or controlling of only a subset of patients was not performed as to avoid selection bias (Table 1).

**Table 1:** Patient profiles of PRP treated and control group

Variable	Control *n* = 1000	PRP *n* = 1000	*p* value
Age	69.7 ± 14	71.5 ± 16	0.0075
Smoker	223	257	0.17
Male/female	811	845	0.55
Body surface area	1.7 ± 0.8	1.9 ± 0.5	0.0001
COPD	192	211	0.41
Chronic renal insufficiency - Dialysis	155	176	0.29
Osteoporosis	118	112	0.79
Oral Steroids	72	93	0.12
Hypertension	659	688	0.55
Diabetes mellitus	267	246	0.43
Ventricular Assist Device/Transplant	96	198	0.0001
CABG with BIMA	11	23	0.056
Emergency Surgery	9	142	0.0018
Urgent Surgery	422	476	0.14
Prior Sternotomy	226	271	0.071
Mean PRBCs transfused	1.5 ± 0.7	1.9 ± 1.5	0.0001
Re-exploration for Bleeding	83	67	0.24
Intubation >24 h	301	344	0.15

*PRP* platelet rich plasma, *COPD* chronic obstructive pulmonary disease, *CABG* coronary artery bypass grafting, *BIMA* bilateral internal mammary arteries, PRBC-packed red blood cells

### Study design and endpoints

All data was collected prospectively and analyzed retrospectively for this consecutive non-randomized group of patients. Readmissions, sternal wound infections and costs were independently verified via the hospital infection committee and financial office. All patients undergoing sternotomy were included in the study.

### Definitions

The diagnosis of DSWI was made, based on the guidelines of the CDC, in patients who developed one or more of the following: [1] positive culture of mediastinal tissue or fluid; [2] clinical evidence of mediastinitis during sternal reoperation; or [3] chest pain, sternal instability, purulent discharge from the mediastinum associated with a positive blood culture.

### Standard sternal closure

All sternotomies were made midline with a sternal saw blade. The sternum was closed with simple interrupted or figure of eight stainless steel wire. The wounds were closed in a layered manner with absorbable suture. The wounds were dressed with Steri-Strips™ (3 M Nexcare, St. Paul, MN), gauze and paper tape. All surgeons at our institution participated in study. Residents and fellows were included in the sternal closure process.

### Platelet rich plasma (PRP) preparation and application

In the treatment population, 52 mL of whole blood was drawn prior to surgery via a central venous line and mixed with 8 mL of the anticoagulant citrate dextrose formula A. The anticoagulated blood (60 mL) was then processed using the Magellan® Autologous Platelet Separator System which is FDA approved to make platelet rich plasma (Arteriocyte Medical Systems, Cleveland, Ohio). The cost of the PRP kit is $385.00. The automated processing time was 15 min which did not require supervision. After this process was finished an audible sound verified completion. There was 6 mL of PRP produced for each patient to be applied to sternum and soft tissue during sternal closure. The analysis of the growth factors is shown in Table 2. The prepared PRP was applied with calcium chloride and thrombin topically onto the exposed sternal edges and the subcutaneous tissue of the chest wound at the time of closure with 6 mL PRP to 1 mL calcium/thrombin ratio. The procedure time for application was 30 s.

**Table 2:** Platelet analysis

Mean ± SD	Baseline (60 mL)	PRP (mL)
PDGF AB (ng/mL)	8.4 ± 2.1	96.1 ± 22.5
PDGF AA (ng/mL)	2.1 ± 0.4	25.4 ± 3.9
PDGF BB (ng/mL)	5.9 ± 1.2	61.3 ± 11.6
TGF B1 (ng/mL)	46.4 ± 4.4	278.2 ± 38.4
VEGF (ng/mL)	76.3 ± 19.5	801 ± 266.1
bFGF (ng/mL)	15.6 ± 2.9	55.1 ± 9.6
EGF (ng/mL)	13.4 ± 2.1	187 ± 29.4

Platelets were analyzed pre and post concentration for growth factors. *PDGF* platelet derived growth factor, *TGF* transforming growth factor, *VEGF* vascular endothelial growth factor, FGF- fibroblast growth factor, *EGF* endothelial growth factor

### Cost analysis

The cost of the PRP procedure included the cost of the disposables. These were actual costs and not hospital charges. These costs were validated by the hospital operating room services director, vendor and hospital finance department. The costs associated with readmission and re-intervention were verified with the hospital operating room services director and the hospital finance department. There was no separate cost allocated to the 30 s for the application of the PRP. Also, the 30 s needed to procure the blood after central venous catheter insertion was not accounted for as the blood draw was included with the standard set of pre-operative laboratory chemistries and other studies.

### Statistical analysis

All patients undergoing open cardiac surgery between January 2005 and January 2013 who met the inclusion criteria were enrolled in the study. The association of the application of PRP, patient care costs and patient outcomes were examined using Fisher’s exact test. Also, the number of patients needed to be treated to see a clinical and cost benefit were calculated.

Ethics, consent and permissions: All procedures performed in studies involving human participants were in accordance with the ethical standards of the institutional and/or national research committee and with the 1964 Helsinki declaration and its later amendments or comparable ethical standards.

## Results

Two thousand consecutive patients completed the study. One thousand of these patients received PRP (PRP group) while the other 1000 patients did not receive PRP (control group). There were significant differences between the two groups with regards to age and body surface area being lower in the control group and more VAD implantations/heart transplants, emergency operations and greater blood transfusion usage in the PRP group. Compared to the control group, the use of PRP reduced the incidence of deep sternal wound infection from 2.0 to 0.6 %, superficial wound drainage from 8.0 to 2.0 %, and the hospital readmission rate within 30 days of operation from 4.0 to 0.8. The time to infection post-surgery demonstrated that all infections in the PRP group occurred in the first 2 months post-surgery whereas in the control group occurred up to 4 months post-surgery Fig. 1. There was a significant reduction in the overall actual cost in the total management of deep and superficial wounds ($1,256,960 in the control group vs $593,791 in the PRP group), Table 3. Despite the significant overall reduction in cost in the management of sternal wounds, the number of patients needed to treat to see a benefit is 71 with 95 % confidence interval 41.8 to 244.5 with a cost of $27,000 to prevent one deep sternal wound infection and cost break-even point. However, with superficial sternal wounds the number of patients needed to treat to see a benefit is 17 with 95 % confidence interval 12.7 to 24.3 with a cost of $6417 to prevent one infection but cost break-even point does not exist. The overall number of patients needed to treat to see a benefit is 14 with 95 % confidence interval 10.5 to 18.9 with a cost of $5203 to prevent one overall wound infection and cost break-even point.

**Table 3:** Results

		Control *n* = 1000	PRP *n* = 1000	*p* value
Infection	Deep Sternal Wound	20	6	0.009
	Superficial Sternal Wound	80	20	0.0001
	Readmission	40	8	0.0001
Cost ($)	Deep Sternal Wound	1,158,840	172,471	0.0001
	Superficial Sternal Wound	98, 120	36,320	0.0087
	Platelet Rich Plasma	0	385,000	0.0001
	Total	1,256,960	593,791	0.012

The number of patients with deep and superficial infections along with the cost of their management including the cost of the platelet rich plasma

## Discussion

Median sternotomy remains the preferred access to the heart for most cardiac surgery operations [13]. Deep sternal wound infection (DSWI) following median sternotomy, however, remains a life threatening complication. The ability to reduce DSWI after an operation as common as open heart surgery has important implications for patients, caregivers, hospitals and payers. While advances in surgical debridement, continuous antibiotic irrigation, flap closure and modern VAC therapy [5–7] have improved DSWI related mortality over the last 30 years, the incidence of sternal wound complications has not changed since the 1980s. As a result of this, the goal would be to prevent to DSWI. There have been a number of approaches including sternal wrapping, vests, and increasing the number of sternal wires used during closure all with limited results [14–16]. There have also been validated risk models to help predict sternal wound issues but not to prevent them [17].

The use of autologous PRP for the prevention of DSWI has been proposed with good clinical results [8]. It is believed that healing following median sternotomy is improved by growth factors released by the PRP. During the inflammatory phase of tissue healing, activated platelets release specific growth factors, such as transforming growth factors-beta, vascular endothelial growth factor and epithelial growth factor (Table 2). These factors stimulate cell proliferation, migration, differentiation, and matrix synthesis. These same factors can affect chondrocyte metabolism, chondrogenesis, and improve bone healing and regeneration [8]. The combination of growth factors contained in the PRP which are released onto the surgical site are the proposed mechanism of action to improved sternal wound healing.

PRP has been used in a number of surgical applications (plastic, maxillofacial and orthopedic surgery) to promote wound and tissue healing [8, 18]. In addition, *Staphylococcus aureus,* the most common bacteria responsible for DSWI can be inhibited by the application of PRP [19]. Several studies have proposed the use of autologous PRP application prior to wound closure in cardiac surgeries with mixed results [20–23]. These studies, although prospective in design, were underpowered to effectively evaluate any treatment effects, due to the small number of patients enrolled. Previous studies report that different methods of producing PRP will result in varying growth factor levels and compositions [24]. Also, the addition of antibiotics to PRP has also been to have positive results but unknown if these are incremental over PRP alone [25]. The use of an automated single device like the one used in this study provides consistent platelet concentration, reproducible results independent of operator and a closed system, reducing the potential for contamination.

This is the first large clinical study to include all patients undergoing median sternotomy independent of emergency status, ventricular assist device implantation, heart transplant, type of surgery, dialysis, steroid use, emphysema, or reoperative surgery. Despite some studies and the Society of Thoracic Surgeons database results reporting DSWI rates as low as 0.3 %, these do not account for all patients undergoing median sternotomy. Most of these studies evaluate elective patients undergoing coronary artery bypass grafting or isolated valve surgery. Also, most studies and/or databases do not report wound outcomes through 6 months post-operatively as our study reports. This would account for the higher wound related infection rates that we report, as up to 40 % of wound complications can occur after 30 days post-surgery [26]. There was also no difference between sternal closure with simple sternal wire or figure of eight sternal wire closure. Also, there were no complications which were attributed to the use of PRP. This study also demonstrates that all infections in the PRP group occur in the first 2 months post-surgery whereas up to 4 months post-surgery in the control group. Even though the study was not powered to validate this finding, it may be inferred that PRP leads to earlier healing of the wound and thus reducing the rate of infection.

The outcomes analysis related to the cost of PRP along with management of sternal wounds is interesting because deep wound infections are so much more expensive than the cost of PRP, once you have prevented one of them, PRP sees a cost advantage. The Number of patients needed to be treated (NNT) and to break even benefit is demonstrated only in the NNT for deep wounds and combined. However, because the cost of treating the superficial wounds is so low, PRP is always more expensive. If the NNT was more like 3.5 instead of 17 for superficial wounds, then PRP would be less costly as soon as the first infection occurred.

There are a number limitations to this study including that it is not a randomized blinded multi-center study. However with the large sample size, the key points are outcomes and costs which can be validated and bring value to the healthcare of cardiac surgery patients. Wound related issues are examined by a number of different healthcare providers including nurse practitioners, physician assistants, primary care physicians, plastic surgeons and cardiologists who are not administering the PRP or primarily part of the study. This helps to minimize bias which can occur if the investigator is always the same person evaluating the wound related issues. The patients were not match as part of this study and the entire population of patients undergoing sternotomy were evaluated. There are a number of confounding variables which may be identified. However with a sample size of 2000 patients and including all surgeons in study along with residents and fellows helping close the sternotomies this provides real world management of cardiac surgery patients. The costs are derived directly from the hospital and financial analysts who are also not directly part of study. As the Centers for Medicaid and Medicare Services do not reimburse for the costs incurred during the treatment of DSWI the findings of this study outweigh its limitations.

## Conclusion

This study demonstrates that the routine use of PRP reduces the incidence of DSWI and SSWI along with the overall costs for management resulting in absolute risk reduction of 7.41 %. For the 2000 patients included in this study, the application of PRP during surgical closure was found to be safe and significantly reduced postoperative infection rates in the treatment group. PRP is a safe, simple and reproducible therapy that appears to provide both a clinical and a financial benefit to patients undergoing sternotomy for cardiac surgery. The addition of PRP to all sternal closure after cardiac surgery brings value by improving care and reducing costs.

### Consent to publish

We have consent to publish de-identified data as per our IRB approval.

## References

[1] Schimmer C, Sommer SP, Bensch M, Leyh R (2007). Primary treatment of deep sternal wound infection after cardiac surgery: a survey of German heart surgery centers. Interact Cardiovasc Thorac Surg.

[2] Ridderstolpe L, Gill H, Granfeldt H, Ahlfeldt H, Rutberg H (2001). Superficial and deep sternal wound complications: incidence, risk factors and mortality. Eur J Cardiothoracic Surg.

[3] Robinson PJ, Billah B, Leder K, Reid CM (2007). ASCTS database committee factors associated with deep sternal wound infection and haemorrhage following cardiac surgery in Victoria. Interact Cardiovasc Thorac Surg.

[4] Tundermann S, Dademasch A, Praetorius J, Kempfert J, Dewey T, Falk V, Mohr FW, Walther T (2011). Comprehensive assessment of frailty for elderly high-risk patients undergoing cardiac surgery. Eur J Cardiothorac Surg.

[5] Fleck TM, Fleck M, Moidl R, Czerny M, Koller R, Giovanoli P, Hiesmayer MJ, Zimpfer D, Wolner E, Grabenwoger M (2002). The vacuum-assisted closure system for the treatment of deep sternal wound infections after cardiac surgery. Ann Thorac Surg.

[6] Douville EC, Asapj JW, Dworkin RJ, Handy JR, Canepa CS, Grunkemeier GL, Wu Y (2004). Sternal preservation: a better way to treat most sternal woun complications after cardiac surgery. Ann Thorac Surg.

[7] Immer FF, Durrer M, Muhlemann KS, Erni D, Gahl B, Carrel TP (2005). Deep sternal wound infection after cardiac surgery: modality of treatment and outcome. Ann Thorac Surg.

[8] Jameson CA (2007). Autologous platelet concentrate for the production of platelet gel. LabMed.

[9] Trowbridge CC, Stammers AH, Woods E, Yen BR, Klayman M, Gilbert C (2005). Use of platelet gel and its effects in infection in cardiac surgery. J Extra Corpor Technol.

[10] Englert SJ, Estep TH, Ellis-Stoll CC (2005). Autologous platelet gel applications during cardiovascular surgery: effect on wound healing. J Extra Corpor Technol.

[11] Vang SN, Brady CP, Christensen KA, Allen KR, Anderson JE, Isler JR, Holt DW, Smith LM (2007). Autologous platelet gel in coronary artery bypass grafting: effects on surgical wound healing. J Extra Corpor technol.

[12] Mangram AJ, Horan TC, Pearson ML, Silver LC, Jarvis WR (1999). Guide-line for prevention of surgical site infection hospital infection control practices advisory committee. Infect Control Hosp Epidemiol.

[13] Massetti M, Babatasi G, Bhoyroo S, Khayat A (1999). The “sternum calvary”. [letter]. J Thorac Cardiovasc Surg.

[14] Kirbas A, Celik S, Gurer O, Yildiz Y, Isik O (2011). Sternal wrapping for the prevention of sternal morbidity in elderly osteoporotic patients undergoing median sternotomy. Tex Heart Inst J.

[15] Celik S, Kirbas A, Gurer O, Yildiz Y, Isik O (2011). Sternal dehiscence in patients with moderate and severe chronic obstructive pulmonary disease undergoing cardiac surgery: the value of supportive thorax vests. J Thorac Cardiovasc Surg.

[16] Kamiya H, Al-maisary SS, Akhyari P, Ruhparwar A, Kallenbach K, Lichtenberg A, Karck M (2012). The number of wires for sternal closure has a significant influence on sternal complications in high-risk patients. Interact Cardiovasc Thorac Surg.

[17] Fischer JP, Basta MN, Wink JD, Nelson JA, Serletti JM, Kovach SJ, Low DW, Vallabhajosyula P, Acker MA, Kanchwala S (2014). An internally validated risk model of deep sternal wound infection (DSWI): a review of 5,179 patients undergoing index median sternotomy procedures. Plast Reconstr Surg.

[18] Dominijanni A, Cristofaro MG, Brescia A, Giudice M (2012). Platelet gel in oral and maxillofacial surgery: a single-centre experience. Blood Transfus.

[19] Bielecki TM, Gazdzik TS, Arendt J, Szczepanski T, Krol W, Wielkoszynski T (2007). Antibacterial effect of autologous platelet gel enriched with growth factors and other active substances-an in vitro study. J Bone Joint Surg (Br).

[20] Buchwald D, Kaltschmidt C, Haardt H, Laczkovics A, Reber D (2008). Autologous platelet gel fails to show beneficial effects on wound healing after saphenectomy in CABG patients. J Extra Corpor technol.

[21] Dorge H, Sellin C, Bury MC, Drescher A, Seipelt R, Grossmann M, Danner BC, Schoendube FA (2013). Incidence of deep sternal would infection is not reduced with autologous platelet rich plasma in high-risk cardiac surgery patients. Thorac Cardiovasc Surg.

[22] Limathe J, Philipp C, Kurt M, Boeken U, Gams E, Feindt P (2009). The use of autologous platelet gel (APG) for high-risk patients in cardiac surgery-is it beneficial?. Perfusion.

[23] Khalafi RS, Bradford DW, Wilson MG (2008). Topical application of autologous blood products during surgical closure following a coronary artyery bypass graft. Eur J Cardiothorac Surg.

[24] Everts PA, Mahoney CB, Hoffmann JJ, Schonberger JP, Box HA, Zundert AZ, Knape JT (2006). Platelet-rich plasma preparation using three devices: implications for platelet activation and platelet growth factor release. Growth Factors.

[25] Hamman BL, Stout LY, Theologes TT, Sass DM, da Graca B, Filardo G (2014). Relation between topical application of platelet-rich plasma and vancomycin and severe deep sternal wound infections after a first median sternotomy. Am J Cardiol.

[26] Mustafa A, Carr C, Alkhafagi S, Mughal N, Omer M, Alkhulaifi A (2014). Late presentation of a deep sternal wound infection and left breast abscess. J Wound Care.

